# Prevalence of opioid adverse events in chronic non-malignant pain: systematic review of randomised trials of oral opioids

**DOI:** 10.1186/ar1782

**Published:** 2005-06-28

**Authors:** R Andrew Moore, Henry J McQuay

**Affiliations:** 1 Pain Research and Nuffield Department of Anaesthetics, University of Oxford, Oxford Radcliffe Hospital, Oxford, UK

## Abstract

Adverse events of opioids may restrict their use in non-cancer pain. Analysis of the incidence of common adverse events in trials conducted in non-cancer pain has usually been limited to opioids used to treat severe pain according to the WHO three-step ladder. To examine the incidence of common adverse events of opioids in non-cancer pain, a systematic review and meta-analysis of information from randomised trials of all opioids in non-cancer pain was undertaken. Studies used were published randomised trials of oral opioid in non-cancer pain, with placebo or active comparator. Thirty-four trials with 5,546 patients were included with 4,212 patients contributing some information on opioid adverse events. Most opioids used (accounting for 90% of patients) were for treating moderate rather than severe pain. Including trials without a placebo increased the amount of information available by 1.4 times. Because of clinical heterogeneity in condition, opioid, opioid dose, duration, and use of titration, only broad results could be calculated. Use of any oral opioid produced higher rates of adverse events than did placebo. Dry mouth (affecting 25% of patients), nausea (21%), and constipation (15%) were the most common adverse events. A substantial proportion of patients on opioids (22%) withdrew because of adverse events. Because most trials were short, less than four weeks, and because few titrated the dose, these results have limited applicability to longer-term use of opioids in clinical practice. Suggestions for improved studies are made.

## Introduction

Opioids are advocated by WHO for cancer pain [1], but their role in chronic non-cancer pain is more controversial. It has been argued that certain types of chronic pain, like neuropathic pain, do not respond to opioids [2], although some patients with neuropathic pain have been shown to respond well to opioids, as do patients with chronic nociceptive pain [3]. Concerns have been expressed about the safety of long-term opioid administration [4] because of adverse effects [5], development of tolerance to the analgesic effect [6], addiction, and drug diversion [7]. Guidelines for responsible use of opioids in chronic non-cancer pain [8-10] reflect concern over these problems.

At least one recent systematic review has investigated efficacy and safety of oral opioids in placebo-controlled randomised trials in chronic non-malignant pain [11]. It included 11 trials in which patients received different oral opioids, doses, and dosing regimens, over varying periods of time, and in patients with arthritis, musculoskeletal pain, neuropathic pain, and mixed conditions. Adverse event rates could be calculated for several adverse events, and numbers needed to harm were calculated, which were as low as 3 for constipation, and 5 for nausea and somnolence. Constipation (affecting 41%), nausea (32%) and somnolence (29%) were the most common adverse events.

The fact that only placebo-controlled trials were included meant that any trials of different opioids, or different dosing regimens, or different formulations that did not have a placebo group were not analysed. The study was additionally limited to opioids (fentanyl, hydromorphone, methadone, morphine, oxycodone, oxymorphone) used to treat severe pain according to the WHO three-step ladder, and did not include other opioids, like codeine, dextropoxyphene, or tramadol, often used to treat moderate pain. The limited number of trials also meant that sensitivity analysis for different conditions (arthritis, musculoskeletal, or neuropathic pain) was not feasible.

To address these points, a further systematic review was conducted to include all randomised double blind trials of any opioid, alone or in combination with other analgesics, irrespective of their being placebo or active controlled. There were several aims. First, to establish how much information was lost if analyses were limited to placebo-controlled trials only. Second, because there was no common comparator for statistical analysis, to establish prevalence rates for oral opioid use in chronic non-malignant pain. Third, to investigate any major differences in opioid adverse events in chronic non-malignant pain of different aetiology.

## Methods

Broad free-text searches were conducted of Medline (1966 to August 2004), EMBASE (1980 to August 2004), Cochrane Library (on-line August 2004) and the Oxford Pain Relief Database (1950 to 1994 [12]), without restriction of language. Reference lists of reports and reviews were also searched. Abstracts, review articles and unpublished reports were not considered. Authors were not contacted for original data.

Inclusion criteria were randomised double blind comparisons of oral opioid with placebo or an active comparator in chronic non-cancer pain. Only double blind studies with at least 10 adult patients completing each treatment arm reporting on adverse events were included. Inclusion was based on a consensus of all reviewers.

QUOROM guidelines for reporting meta-analyses were followed [13]. Each report was scored for quality and validity using a three-item (1 to 5) quality scale [14] assessing the quality of randomisation, double-blinding and reporting on withdrawals and dropouts. The minimum score for inclusion was 2 points (one each for randomisation and blinding).

Information about treatments and controls, numbers randomised and analysed, type and dose of opioid, and duration of study was extracted. Information about adverse events was looked for and extracted, particularly the number of patients experiencing any adverse event, withdrawing because of adverse events or lack of efficacy, and patients experiencing particular adverse events.

There was prior intent to analyse data for all patients, irrespective of condition, and to analyse separately for patients with arthritis (any site), other musculoskeletal conditions (back pain, for instance), neuropathic pain, and pain of mixed origin. No statistical analysis was planned, as it was considered unlikely that there would be sufficient consistency of drug used, dose, titration regimen, duration, or comparator that would provide sufficient information for any sensible comparator. It was planned to estimate the prevalence of adverse events for patients receiving oral opioids or placebo, both overall and for each condition. Adverse event rates would be calculated with a 95% confidence interval.

## Results

Searches found 35 trials with a total of 5,546 patients, although one trial [15] had no interpretable data and contributed no patients to the analysis. Most trials and patients were in arthritis or musculoskeletal conditions, with few patients with neuropathic pain, and some in mixed nociceptive and neuropathic conditions (Table 1). All but one trial had quality scores of 3 or more out of 5. Twenty-eight trials were of one to four weeks. Four were shorter (three to six days) and two were longer (one of 30 days, and one of 8 weeks). Adverse events were not usually recorded using formal diaries (three trials) or questionnaires (two trials). Most trials either collected volunteered information, or asked general questions about adverse events. Detailed information on each trial, together with the references of the trials included, is in Additional file 1.

The number of patients given different opioids in each condition, and overall, is shown in Table 2. Overall, 4,212 patients contributed some data on opioid adverse events. Just over half the patients received tramadol or tramadol plus paracetamol, and combinations of codeine or dextropropoxyphene with paracetamol contributed another quarter. Other than tramadol, morphine was the only opioid to have been tested in each condition. Only 10% of patients received oral opioids (morphine or oxycodone) to treat severe pain. Some form of initial dose titration was mentioned in 14 of the 34 trials. Dose titration was uncommon in arthritis or musculoskeletal conditions (3/16 trials in arthritis, 4/10 trials in musculoskeletal pain), but very common in trials involving neuropathic pain (4/5) or pain of mixed origin (3/3).

Of the 34 trials contributing data to the analysis, 16 (47%) had a placebo control. Of the 5,546 patients, 2293 (41%) were in trials that had a placebo control. The frequency of placebo control was highest in neuropathic pain studies, with four of five trials and 87% of patients. The policy of including trials without a placebo increased the potential amount of information by about 1.4 times. Other comparators used included paracetamol, diclofenac, and nortriptyline, each in a single trial.

**Figure 1:**
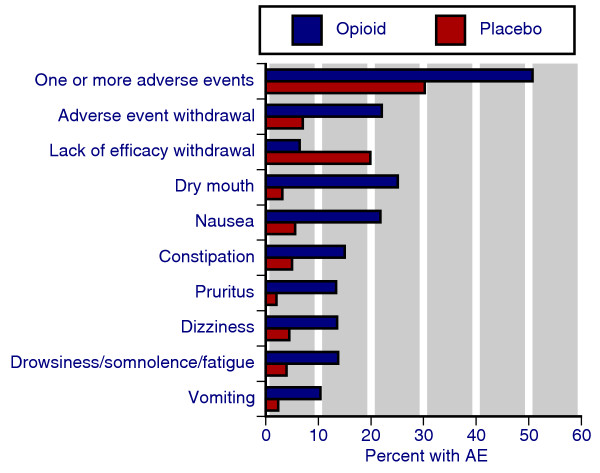
Adverse event (AE) rates with opioid and placebo in chronic non-malignant pain.

The overall rates of patients experiencing any adverse event, adverse event or lack of efficacy withdrawal, or particular adverse events for both opioid and placebo are shown in Table 3 and Fig. 1. Event rates were higher for opioids than placebo for all adverse events, with the exception of lack of efficacy withdrawal, when placebo was higher than opioid.

In these relatively short-term trials, about half the patients would experience at least one adverse event, and about one in five would stop taking opioids because of the adverse event. With opioids, the adverse events reported were those likely to be encountered with the use of opioids, namely nausea or vomiting, constipation, drowsiness and dizziness, all of which were commonly reported in large samples of between 3,000 and 4,300 patients. Dry mouth and pruritus were reported in relatively small numbers of patients in relatively small samples. Event rates for particular adverse events were between 10% and 25% (Table 3). Information was available from much fewer patients receiving placebo. Typically, only a few tens of patients out of several hundred enrolled in the trials reported particular adverse events. For individual adverse events only about 2% to 5% of patients were affected (Table 3).

These average results conceal very great variation between individual trials. For instance, constipation in individual trials could be reported in as few as 0%, and as high as 71%, and nausea between 5% and 98% (Additional file 2). These large variations typically were found in the smaller trials, and there was no obvious relationship between high or low rates and drug, dose, or dosing regimen.

**Figure 2:**
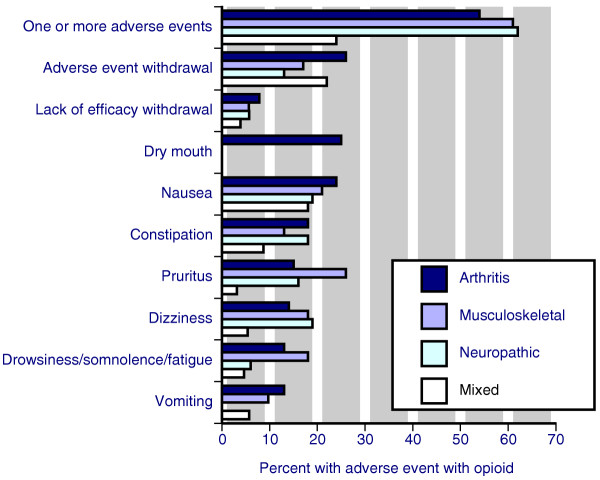
Adverse event rates in patients with different aetiology of chronic non-malignant pain treated with opioids.

Different painful conditions produced generally similar patterns of results, and Additional file 2 reports the details of the number of trial arms, patients, average prevalence and range of results found in individual trials. Fig. 2 shows the comparison pictorially. Event rates for each adverse event were consistent for all four conditions, although there was a tendency to lower adverse event rates in trials in mixed conditions, and to some extent in neuropathic pain, where more trials used dose titration rather than fixed dosing schedules.

## Discussion

This systematic review is different from two previous reviews of opioids in chronic non-malignant pain [11,16] in that it accepted trials of any opioid rather than only trials of opioids used to threat severe pain on the WHO ladder (morphine, oxycodone, fentanyl). More than 90% of the 4,212 patients received oral opioids not usually used to treat severe pain on the WHO three-step ladder. Because this review includes trials that did not have a placebo comparator, information on adverse events was available from many more patients. In total, information from 34 randomised trials with 5,546 patients was available (Table 1), much more information than available in previous reviews with 11 randomised trials and 1025 patients [11] or 16 randomised trials with 1,427 patients [16]. The policy of including studies without a placebo comparator meant that 1.4 times more information was available.

The trials included were of good reporting quality, with all but one of the 34 contributing data having quality scores of 3 or more out of the maximum of 5. For efficacy analysis, this level of reporting quality is not likely to suffer bias associated with lack of randomisation or blinding. Trials were of relatively short duration, however, limiting their utility. Only two lasted longer than four weeks, and only one was as long as eight weeks. It is possible that this might limit how general the results might be, as some tolerance to adverse events with opioids with longer use is expected.

It could be argued that for most people with chronic non-malignant pain for whom opioids are an option, oral opioids other than morphine or oxycodone would be preferable. Here, three-quarters of the information on opioids was in patients receiving codeine or tramadol, alone or in combination with paracetamol.

The great variety of opioid drugs used, with different doses, different dosing schedules, different comparators, and in different conditions meant that no statistical analysis was possible. Many of the trials were small, so that the potential for large effects from the random play of chance could not be excluded [17]. Methods used to collect adverse event information also varied, with few studies using diaries or questionnaires to obtain information in a systematic way. Even in acute pain studies, the method of adverse event ascertainment can influence measured adverse event prevalence [18]. Less than adequate reporting of adverse event information in clinical trials is relatively common [19]. Randomised trials may not detect all adverse events, especially in small short-term studies. In these circumstances, trying to draw any conclusions by comparing one trial with another would not be prudent.

Because the literature on opioid use in chronic non-malignant pain was known to be clinically heterogeneous, the prior intent was only to provide overall prevalence for adverse events with oral opioids in a general way. Several thousand patients contributed information for many adverse events with opioids, with the exception of pruritus and dry mouth, which were less frequently reported (Table 3). About one patient in two experienced at least one adverse event, and one in five discontinued because of adverse events. Nausea (21%), constipation (15%), dizziness (14%), and drowsiness or somnolence (14%) were the most common particular adverse events. This was similar in order but lower in frequency than that found previously with other opioids in chronic non-malignant pain (constipation, 41%; nausea, 32%; and somnolence, 29%) [9]. Adverse event rates were similar in four separate clinical conditions of arthritis pain, musculoskeletal pain, neuropathic pain, and pain of mixed nociceptive and neuropathic origin. Somewhat lower adverse event rates in the latter categories might have been associated with a higher use of dose titration.

Adverse events with opioids were clearly higher than with placebo (Fig. 1, Table 3), as expected. Rates of adverse events with placebo were not unlike those found in young healthy individuals in the USA and Germany, three decades apart [20,21], where fatigue (37% to 65%), pain in muscles and joints (5% to 13%), dry mouth (3% to 5%), constipation (3% to 4%), nausea (1% to 3%) and vomiting (0% to 2%) were not infrequent. Many other symptoms were mentioned in high frequency. A large systematic review of constipation prevalence studies in North America [22] found rates between about 2% and 27%, but mostly about 15%, considerably higher than the 5% found for placebo in the clinical trials, and the same as for patients receiving opioids for chronic non-malignant pain (Table 3).

Future trials should consider a number of crucial variables, including dose. The higher frequency of adverse events with opioids used to treat severe pain [11] than with opioids more often used to treat moderate pain found in this review, but with similar rank order, may be explained by higher dose equivalents with the former. Duration is also important. Patients starting on opioids are usually told to expect initial adverse events, such as nausea and drowsiness, but that these will improve rapidly. Judging the adverse event profile of long-term opioid use from short-term trials is unlikely to be satisfactory for extrapolation to longer-term use in clinical practice.

Similarly, the naivety of the patients to opioids may be an issue in adverse event incidence, and we were unable to analyse this data set by previous opioid exposure. In real clinical life as opposed to trials, dose is usually titrated. That was the case in some of these trials, particularly in neuropathic pain, but not many. We were unable to test whether adverse effects incidence was different between trials with dose titration and trials using a fixed dose. All of these points are relevant for the design and conduct of future trials.

## Conclusion

The review demonstrates that randomised trials of opioids in chronic non-malignant pain have been predominantly of a short duration, arguably too short to satisfy modern requirements of trial design in chronic pain. Adverse event information must therefore be treated with a degree of caution. However, information from large numbers of patients confirm that most patients will experience at least one adverse event, and that substantial minorities will experience common opioid adverse events of dry mouth, nausea, and constipation, and will not continue treatment because of intolerable adverse events.

## Competing interests

RAM and HJM have received lecture fees from pharmaceutical companies. The authors have received research support from charities and government sources at various times. This work was supported by an unrestricted educational grant from Pfizer Ltd. The terms of the financial support from Pfizer included freedom for authors to reach their own conclusions, and an absolute right to publish the results of their research, irrespective of any conclusions reached. Pfizer did have the right to view the final manuscript before publication, and did so. Neither author has any direct stock holding in any pharmaceutical company.

## Authors' contributions

RAM was involved with planning the study, data extraction, analysis and preparing a manuscript; HJM with planning, analysis and writing. Both authors read and approved the final manuscript.

## Supplementary Material

Additional File 1 A pdf file which contains details of individual studies. Click here for file

Additional File 2 A pdf file which lists the adverse events with opioids in different pain conditions. Click here for file

## Figures and Tables

**Table 1:** Randomised double blind trials of opioids in chronic non-malignant pain of different aetiology

	Number of			Number of	
Condition	Trials	Patients	Drug treaments	Trial arms	Contributing patients
Arthritis	16	2990	Opioid	24	2173
			Placebo	7	445
			Other comparator	2	447
Musculoskeletal	10	1648	Opioid	18	1561
			Placebo	4	302
			Other comparator	0	0
Neuropathic	5	302	Opioid	5	219
			Placebo	4	178
			Other comparator	1	46
Mixed conditions	3	606	Opioid	6	606
			Placebo	1	49
			Other comparator	0	0

**Table 2:** Opioids used in trials of non-malignant pain of different aetiology

	Condition^a^				
Drug	Arthritis	Musculoskeletal	Neuropathic	Mixed	All conditions

Codeine	51	46			97
Codeine + paracetamol	173	233		156	562
Dihydrocodeine	119				119
Dextropropoxyphene	129	56			185
Dextropropoxyphene + paracetamol	496				496
Morphine	222	61	59	49	391
Meptazinol		31			31
Oxycodone		47	50		97
Pentazocine	76				76
Tramadol	363	778	110	401	1652
Tramadol + paracetamol	197	309			506
Total	1826	1561	219	606	4212

^a^Values are numbers of patients treated with the different opioids.

**Table 3:** Adverse events with oral opioid and placebo in randomised trials in chronic non-malignant pain

	Oral opioid				Placebo			
	Number of patients		Event rate (%)		Number of patients		Event rate (%)	

Adverse event	With AE	Total	Average	95% CI	With AE	Total	Average	95% CI

Patients experiencing any adverse event	1387	2743	51	49–53	122	404	30	26–34
Withdrawals								
Adverse event withdrawal	933	4221	22	21–23	51	721	7.1	5.2–8.9
Lack of efficacy withdrawal	177	2719	6.5	5.6–7.4	135	679	20	17–23
Specific adverse events								
Dry mouth	100	399	25	21–29	3	94	3.2	0–6.7
Nausea	902	4314	21	20–22	40	718	5.6	3.9–7.2
Constipation	606	4043	15	14–16	32	643	5.0	3.3–6.7
Dizziness	530	3899	14	13–15	28	623	4.5	2.9–6.1
Drowsiness or somnolence	425	3078	14	13–15	21	531	4.0	2.3–5.6
Pruritus	106	794	13	11–16	7	336	2.1	0.6–3.6
Vomiting	345	3330	10	9.3–11	12	497	2.4	1.1–3.8

AE, adverse event; CI, 95% confidence interval.
