# Supplementary material for: Prevalence of opioid adverse events in chronic non-malignant pain: systematic review of randomised trials of oral opioids
Source: Arthritis Res Ther. 2005 Jun 28;7(5):R1046–51. doi: 10.1186/ar1782 (PMC1257433; doi:10.1186/ar1782)
Supplement: Additional File 1 — A pdf file which contains details of individual studies. [file ar1782-S1.pdf]

Appendix 1: Details of individual studies

Reference	Drug	Study	Methods	Duration of study / studies	Dose and number of patients	Patients with any adverse event(s)	Patients with particular adverse events	Withdrawal because of adverse events	Withdrawal because of lack of efficacy
Arthritis									
Adler L, McDonald C, O'Brien C, Wilson M. A comparison of once-daily tramadol with normal release tramadol in the treatment of pain in osteoarthritis. J Rheumatol 2002; 29(10):2196-9.	Tramadol (once daily)<br><br>Tramadol (normal release)	Clinical trial	Random, double blind (double dummy), placebo controlled, active controlled, parallel group, OA. Excluded users or monoamine oxidase inhibitors within 2 wks, 1 wk for long-acting NSAIDs. No other pain medicines allowed. Moderate to severe baseline pain intensity. Volunteered adverse events were collected; severity rated by investigator<br>QS=4	7-10 day titration<br><br>4 wk assessment period	T once daily<br>150 to 400 mg (n=188)<br><br>T normal release<br>50 mg tds, 50 mg qds, 100 mg tds, 100 mg tds (n=91)<br><br>Doses titrated over 7-10 days<br><br>Rescue med: paracetamol 1000 mg, max allowed 4000 mg daily	T od 72/188 (38%)<br>T nr 32/91 (35%)	Constipation<br>T od 43/188 (23%)<br>T nr 28/91 (31%)<br>Dizziness<br>T od 38/188 (20%)<br>T nr 15/91 (17%)<br>Drowsiness<br>T od 28/188 (15%)<br>T nr 2/91 (24%)<br>Nausea<br>T od 68/188 (36%)<br>T nr 33/91 (36%)<br>Vomiting<br>T od 36/188 (19%)<br>T nr 16/91 (18%)	Withdrawal because of adverse events<br>T od 69/188 (37%)<br>T nr 32/91 (35%)<br><br>Withdrawal because of adverse events & lack of efficacy<br>T od 5/188 (3%)<br>T nr 4/91 (4%)	T od 16/188 (9%)<br>T nr 8/91 (9%)
Anonymous. Two analgesics compared in osteoarthrosis. Practitioner 1972; 208:557-60.	Dihydrocodeine<br><br>Pentazocine HCl	Clinical trial	Random, double blind, active controlled, cross-over trial, OA. Monoamine oxidase inhibitors excluded. Baseline pain was moderate to severe. Adverse events were recorded<br>QS=2	3 wks each cross-over	N=76<br><br>Dihydrocodeine 30 mg<br><br>Pentazocine 50 mg<br><br>Doses: 1-2 tablets x 4 daily	DHC 52/76 (68%)<br>Pentaz 48/76 (63%)	Dizziness<br>DHC 9/76 (12%)<br>Pentaz 12/76 (16%)<br>Drowsiness<br>DHC 4/76 (5%)<br>Pentaz 5/76 (5%)<br>Nausea<br>DHC 12/76 (16%)<br>Pentaz 10/76 (13%)<br>Vomiting<br>DHC 12/76 (16%)<br>Pentaz 9/76 (12%)<br>Constipation<br>DHC 8/76 (11%)<br>Pentaz 0/76 (0%)	DHC 52/76 (68%)<br>Pentaz 48/76 (63%)	No data
Boissier C et al. Acceptability and efficacy of two associations of paracetamol with a central analgesic (dextropropoxyphene or codeine): comparison in osteoarthritis. J Clin Pharmacol 1992; 32:990-5.	Dextropropoxyphene plus paracetamol<br><br>Codeine plus paracetamol	Clinical trial	Random, double blind (double dummy), placebo controlled, active controlled, parallel group, OA. 2 day washout. No other pain medicines allowed. No criteria for baseline pain intensity, but VAS scores were 60-65 mm. Adverse events collected using questionnaire<br>QS=5	6 days	Dextropropoxyphene 30 mg plus paracetamol 400 mg per capsule (n=71)<br><br>Codeine 30 mg plus paracetamol 500 mg per tablet (n=70)<br><br>6 tablets / capsules given daily	DP + P 50/71 (70%)<br>C + P 60/70 (82%)	Gastrointenstinal<br>DP + P 42/71 (59%)<br>C + P 53/70 (76%)<br>Neurological<br>DP + P 21/71 (30%)<br>C + P 44/70 (63%)	DP + P 9/71 (13%)<br>C + P 27/70 (39%)<br><br>Nausea, vomiting, abdominal pain, vertigo<br><br>None serious enough to cause admission to hospital	1 patient group not stated
Boureau F et al. Placebo-controlled study of the analgesic efficacy of a combination of paracetamol and codeine in rheumatoid arthritis. ACTA THER 1991; 17:123-36.	Codeine plus paracetamol<br><br>Placebo	Clinical trial	Random, double blind, placebo controlled, RA, parallel group. Baseline pain moderate or severe. All other pain medications were discontinued. No washout before study entry. All other pain medicines were stopped. Adverse events collected in patient diaries<br>QS=3	7 days	Cod 30 mg + para 500 mg tid (n=20)<br><br>Placebo (n=20)	C + P 10/20 (50%)<br>Placebo 6/20 (30%)	Constipation<br>C + P 6/20 (30%)<br>Placebo 1/20 (5%)<br>Nausea<br>C + P 3/20 (15%)<br>Placebo 1/20 (5%)<br>Vomiting<br>C + P 2/20 (10%)<br>Placebo 0/20 (0%)	C + P 2/20 (10%)<br>Placebo 3/20 (15%)	C + P 0/20 (0%)<br>Placebo 2/20 (10%)

Caldwell JR, Hale ME, Boyd RE et al. Treatment of osteoarthritis pain with controlled release oxycodone or fixed combination oxycodone plus acetaminophen added to nonsteroidal antiinflammatory drugs: a double blind, randomized, multicenter, placebo controlled trial. J Rheumatol 1999; 26(4):862-9.	Oxycodone controlled release<br><br>Oxycodone immediate release<br><br>Oxycodone plus paracetamol<br><br>Placebo<br><br>In addition to normal NSAID therapy	Clinical trial	Random, double blind (double dummy), active & placebo controlled, parallel group, **add-on** study, OA. All other pain medications were discontinued. All patients continued NSAID therapy at pre-study doses, oral steroids allowed if dose stable for 1 mth. Titration phase to fix dose required (pain less than moderate), then randomisation. Adverse events collected<br>QS=5<br><br>**NB:** Titration phase before randomisation implies responders only continued into DB phase. Enriched enrolement?	30 days	Titration phase (30 days): Oxy IR 5 mg x 4 daily, max allowed 60 mg.<br><br>Double blind treatments:<br>Oxy CR 10 mg x 2 daily (n=34)<br><br>Oxy IR 5 mg plus para 325 mg x 4 daily (n=37)	No data	Pts with drug-related AEs<br>Constipation<br>O CR 24/34 (71%)<br>O IR 20/37 (54%)<br>Dizziness<br>O CR 4/34 (12%)<br>O IR 9/37 (24%)<br>Dry mouth<br>O CR 11/34 (32%)<br>O IR 20/37 (54%)<br>Nausea<br>O CR 5/34 (15%)<br>O IR 14/37 (38%)<br>Pruritus<br>O CR 11/34 (32%)<br>O IR 14/37 (38%)<br>Vomiting<br>O CR 2/34 (6%)<br>O IR 4/37 (11%)	O CR 24/34 (71%)<br>O IR 20/37 (54%)	O CR 3/34 (10%)<br>O IR 4/37 (11%)
Caldwell JR, Rapoport RJ, Davis JC et al. Efficacy and safety of a once-daily morphine formulation in chronic, moderate-to-severe osteoarthritis pain: results from a randomized, placebo-controlled, double-blind trial and an open-label extension trial. J Pain Symptom Manage 2002; 23(4):278-91.	Morphine CR once daily<br><br>Morphine 15 mg IR, twice daily<br><br>Placebo	Clinical trial	Random, double blind (double dummy), active controlled, parallel group, OA. 2 day washout. No other pain medicines allowed. 7 day washout. Baseline pain intensity moderate to severe. Adverse events collected including severity & relation to drug.<br>171/295 pts were opiate naive at study entry<br>QS=5	4 wks	Morphine CR 30 mg once daily, morning (n=73)<br><br>Morphine CR 30 mg once daily, evening (n=73)<br><br>Morphine IR 15 mg, twice daily (n=76)<br><br>Placebo (n=73)	No data	Constipation<br>M CR, am 36/73 (49%)<br>M CR, pm 29/73 (40%)<br>M IR 22/76 (29%)<br>Placebo 3/73 (4%)<br>Dizziness<br>M CR, am 7/73 (10%)<br>M CR, pm 7/73 (10%)<br>M IR 9/76 (12%)<br>Placebo 1/73 (1%)<br>Dry mouth<br>M CR, am 4/73 (6%)<br>M CR, pm 3/73 (4%)<br>M IR 2/76 (3%)<br>Placebo 1/73 (1%)<br>Nausea<br>M CR, am 15/73 (21%)<br>M CR, pm 23/73 (32%)<br>M IR 20/76 (26%)<br>Placebo 7/73 (10%)<br>Somnolence<br>M CR, am 12/73 (16%)<br>M CR, pm 9/73 (12%)<br>M IR 9/76 (12%)<br>Placebo 0/73 (0%)<br>Vomiting<br>M CR, am 473 (6%)<br>M CR, pm 12/73 (16%)<br>M IR 6/76 (8%)<br>Placebo 1/73 (1%)	M CR, am 17/73 (23%)<br>M CR, pm 18/73 (25%)<br>M IR 18/76 (24%)<br>Placebo 5/73 (7%)<br><br>Serious AE:<br>6 pts, 1 hospitalised for constipation.	M CR, am 9/73 (12%)<br>M CR, pm 12/73 (16%)<br>M IR 8/76 (11%)<br>Placebo 14/73 (19%)
Doak W et al. A novel combination of ibuprofen and codeine phosphate in the treatment of osteoarthritis: A double-blind placebo controlled study. J DRUG DEV 1992; 4:179-87.	Codeine<br><br>Placebo<br><br>Also ibuprofen plus codeine, and ibuprofen alone	Clinical trial	Random, double blind, placebo controlled, crossover, OA. All non study drugs were discontinued. Paracetamol 500-4000 mg allowed daily for breakthrough pain uncontrolled by study drugs. no criteria for baseline pain. Adverse events were volunteered, with severity ratings.<br>QS=3	7 days each treatment	Actual doses of drugs taken is not specifically stated.				

Jensen EM et al. Tramadol versus dextropropoxyphene in the treatment of osteoarthritis: A short term double-blind study. DRUG INVEST 1994; 8:211-8.	Tramadol<br><br>Dextropropoxyphene napsylate 100 mg (100 mg napsylate is equivalent to 65 mg hydrochloride)	Clinical trial	Random, double blind, active control, parallel groups, OA hip or knee, moderate to severe pain at baseline.3-7 day washout with paracetamol 4000 mg daily only allowed.Direct questionning about adverse events.<br>QS=5	2 wks	Tramadol 100 mg x 3 daily (n=135)<br><br>Dextropropoxyphene napsylate 100 mg x 3 daily (n=129)	Tram 75/135 (56%)<br>DP 41/129 (32%)	Constipation<br>Tram 11/135 (8%)<br>DP 10/129 (8.5%)<br>Dizziness<br>Tram 23/135 (17%)<br>DP 6/129 (5%)<br>Nausea<br>Tram 35/135 (26%)<br>DP 13/129 (10%)<br>Vomiting<br>Tram 23/135 (17%)<br>DP 3/129 (2%)	Tram 48/135 (36%)<br>DP 14/129 (11%)	No data
Kjaersgaard-Andersen P, Nafei A, Skov O et al. Codeine plus paracetamol versus paracetamol in longer-term treatment of chronic pain due to osteoarthritis of the hip. A randomised, double-blind, multi-centre study. Pain 1990; 43(3):309-18.	Codeine plus paracetamol<br><br>Paracetamol	Clinical trial	Random, double blind, placebo controlled, parallel group. OA hip. Rescue medication: ibuprofen 400 mg (max allowed 1 tablet 3 times daily)<br>All other pain medications were discontinued. No washout before study entry. Adverse events collected using diaries (no checklist) with nonspecific question "did the medicine cause you any discomfort?'<br>Baseline PI mild in 10/83 and 7/75. Compliance was greater than 75%<br>QS=4	4 wks	Cod 60 mg + Para 1000 mg 3 times daily (n=83)<br><br>Paracetamol 1000 mg 3 times daily (n=75)	None were serious<br>C + P 72/83 (87%)<br>P 28/74 (38%)	Constipation<br>C + P 17/83 (20%)<br>P 7/74 (10%)<br>Dizziness<br>C + P 26/83 (31%)<br>P 1/74 (1%)<br>Diarrhoea<br>C + P 8/83 (10%)<br>P 5/74 (7%)<br>Nausea<br>C + P 34/83 (41%)<br>P 6/74 (8%)<br>Somnolence<br>C + P 14/83 (17%)<br>P 5/74 (7%)<br>Vomiting<br>C + P 19/83 (23%)<br>P 3/74 (4%)	C + P 40/83 (48%)<br>P 10/74 (14%)	8 patients (not stated which groups)
Lloyd RS, Costello F, Eves MJ, James IG, Miller AJ. The efficacy and tolerability of controlled-release dihydrocodeine tablets and combination dextropropoxyphene/paracetamol tablets in patients with severe osteoarthritis of the hips. Curr Med Res Opin 1992; 13(1):37-48.	Dihydrocodeine controlled release<br><br>Dextropropoxyphene plus paracetamol	Clinical trial	Random, double blind, active controlled, parallel group. General practice. Severe OA hip. Adverse events collected with checklist & Volunteeredby patients. Severity recorded as mild, moderate, severe<br>Patients previously on NSAIDs for >2 wks were allowed to continue taking them at unchanged doses<br>QS=4	2 wks	CR DHC 60 mg (n=43)<br>(1-2 tablets twice daily)<br>Daily dose DHC 120 to 240 mg<br><br>DP 65 mg + para 650 mg (n=43)<br>(3-4 times daily).<br>Daily dose DP 195 or 260 mg plus P 1950 to 2600 mg	No data	Nausea or vomiting<br>CR DHC 18/43 (42%)<br>DP + P 10/43 (23%)<br>Constipation<br>CR DHC 10/43 (23%)<br>DP + P 10/43 (23%)<br>Drowsiness<br>CR DHC 15/43 (35%)<br>DP + P 13/43 (30%)<br>Difficulty concentrating<br>CR DHC 7/43 (16%)<br>DP + P 11/43 (26%)<br>Dry mouth<br>CR DHC 28/43 (65%)<br>DP + P 30/43 (70%)	CR DHC 1/43 (2%)<br>DP + P 2/43 (5%)	CR DHC 17/43 (40%)<br>DP + P 4/43 (10%)
Parr G et al. Joint pain and quality of life; results of a randomised trial. Br J Clin Pharmacol 1989; 27:235-42.	Dextropropoxyphene plus paracetamol<br><br>Diclofenac SR	Clinical trial	Random, double blind, active control, parallel groups, joint pain.<br>QS=4	4 wks	Dextropropoxyphene plus paracetamol (distalgesic), 2 tablets given x3 daily (n=382)<br><br>Diclofenac SR x 1 daily (n=373)	No data	Constipation<br>DPP 8/382 (2%)<br>Diclo SR 7/373 (2%)<br>Dizziness<br>DPP 30/382 (8%)<br>Diclo SR 14/373 (14%)<br>Nausea<br>DPP 33/382 (9%)<br>Diclo SR 24/373 (6%)	DPP 42/382 (11%)<br>Diclo SR 39/373 (10%)	DPP 8/382 (2%)<br>Diclo SR 5/373 (1%)

Peloso PM, Bellamy N, Bensen W et al. Double blind randomized placebo control trial of controlled release codeine in the treatment of osteoarthritis of the hip or knee. J Rheumatol 2000; 27(3):764-71.	Codeine controlled release<br><br>Placebo	Clinical trial	Random, double blind, placebo control, parallel groups, OA hip or knee, moderate to severe flare pain at baseline.after a 2-7 day washout. No non-study medicines, other than paracetamol 650 mg three times daily, were allowed. Adverse events collected using diaries. 7 pts in each group had previously used long-term codeine<br>QS=5	4 wks	Codeine 100 mg controlled release x 2 daily (n=51)<br><br>Placebo (n=52)	C cr 100 mg 42/51 (82%)<br>Placebo 30/52 (58%)<br><br>Severe adverse events:<br>C cr 100 mg 7/51 (14%)<br>Placebo no data	Constipation<br>C cr 100 mg 25/51 (49%)<br>Placebo 6/52 (11%)<br>Dizziness<br>C cr 100 mg 17/51 (33%)<br>Placebo 4/52 (8%)<br>Somnolence<br>C cr 100 mg 20/51 (39%)<br>Placebo 5/52 (10%)<br>Nausea<br>No significant difference between groups	C cr 100 mg 15/51 (29%)<br>Placebo 4/52 (8%)	C cr 100 mg 1/51 (2%)<br>Placebo 5/52 (10%)
Roth SH et al. Around-the-clock, controlled-release oxycodone therapy for osteoarthritis-related pain. Arch Intern Med 2000;160:853-860.	Oxycodone Controlled Release<br><br>Placebo	Clinical trial	Random, double blind, parallel groups, placebo control. Osteoarthritis pain ≥ 1yr; pain moderate to severe. Excluded history of drug/alcohol abuse. Patients could continue on NSAIDs (65%), no additional analgesics allowed. Previous opioids 61%<br>QS=4	14 days	Oxycodone CR 10 mg x 2 (n=44)<br><br>Oxycodone CR 20 mg x 2 (n=44)<br><br>Placebo (n=45)<br><br>Final daily dose 20 mg or 40 mg<br>mean daily dose 40 mg	87/133 (65%) patients, group not stated<br><br>None were life-threatening.	Treatment related adverse events<br><br>Constipation<br>O CR 20 mg 10/44 (23%)<br>O CR 40 mg/d 13/44 (32%)<br>Placebo 3/45 (7%)<br>Dizziness<br>O CR 20 mg 13/44 (30%)<br>O CR 40 mg 9/44 (20%)<br>Placebo 4/45 (9%)<br>Nausea<br>O CR 20 mg 12/44 (27%)<br>O CR 40 mg 18/44 (41%)<br>Placebo 5/45 (11%)<br>Vomiting<br>O CR 20 mg 5/44 (11%)<br>O CR 40 mg 10/44 (23%)<br>Placebo 3/45 (7%)<br>Pruritus<br>O CR 20 mg 8/44 (18%)<br>O CR 40 mg 7/44 (16%)<br>Placebo 1/45 (2%)<br>Somnolence<br>O CR 20 mg 11/44 (25%)<br>[illegible]	O CR 20 mg 12<br>O CR 40 mg 14<br>Placebo 2	O CR 20 mg12<br>O CR 40 mg 5<br>Placebo 22
Roth SH. Efficacy and safety of tramadol HCl in breakthrough musculoskeletal pain attributed to osteoarthritis. J Rheumatol 1998; 25(7):1358-63.	Tramadol HCl<br><br>Placebo	Clinical trial	Random, double blind, placebo control, parallel groups, **add-on design**, OA hip or knee, NSAID therapy continued. Included mild flare pain. Open lable run-in with normal NSAID therapy plus tramadol 200 mg then 50 mg every 6 hrs (250 mg max on day 1). **Patients willing to continue were randomised** to double blind tramadol or placebo in addition to NSAID for 13 days. Patients recorded adverse events. 17% had moderate/severe pain at randomisation<br>QS=3	13 days	Tramadol HCl 50-100 mg every 4-6 hrs (max 400 mg daily) (n=20)<br><br>Placebo (n=21)<br><br>Average daily dose (double blind): 5 capsules, 250 mg	No data	Constipation<br>Tram 9/20 (45%)<br>Placebo 0/21 (0%)<br>Dizziness<br>Tram 3/20 (15%)<br>Placebo 0/21 (0%)<br>Drowsiness<br>Tram 5/20 (25%)<br>Placebo 3/21 (14%)<br>Dry mouth<br>Tram 2/20 (10%)<br>Placebo 2/21 (10%)<br>Nausea<br>Tram 7/20 (35%)<br>Placebo 3/21 (14%)<br>Vomiting<br>Tram 2/20 (10%)<br>Placebo 2/21 (10%)	Open label phase:<br>13/65 patients discontined because of adverse events<br><br>Double blind phase:<br>Tram 1/20 (5%)<br>Placebo 5/21 (25%)	Tram 3/20 (15%)<br>Placebo 8/21 (38%)

Schnitzer TJ et al. Tramadol allows reduction of naproxen dose among patients with naproxen-responsive osteoarthritis pain: a randomized, double-blind, placebo-controlled study. Arthritis & Rheumatism 1999; 42:1370-7.	Tramadol<br><br>Placebo	Clinical trial	Random, double blind, placebo control, parallel groups, **add-on design**, OA. Moderate to severe pain after 1 wk washout. 5 wk open label run-in with naproxen 500 mg daily for 1 wk. if pain intensity >20 mm on VAS patients were given 1000 mg daily for 3 wks; if lower they discontinued. In third wk, patients were also given tramadol 200 mg daily. Then randomised to double blind tramadol or placebo in addition to naproxen for 8 wks. Dose of naproxen was reduced from 750 mg, by 250 mg every 2 wks. Adverse events collected by spontaneous reporting & non-specific questioning QS=3	8 wks	Tramadol 200 mg daily (n=117)<br><br>Placebo (n=123)	No data for double blind phase only	No data for double blind phase only	T200 mg 26/117 (22%)<br>Placebo 16/123 (13%)	No data for double blind phase only
Silverfield JC, Kamin M, Wu SC, Rosenthal N. Tramadol/acetaminophen combination tablets for the treatment of osteoarthritis flare pain: a multicenter, outpatient, randomized, double-blind, placebo-controlled, parallel-group, add-on study. Clin Ther 2002; 24(2):282-97.	Tramadol plus acetaminophen	Clinical trial	Random, double blind, placebo controlled, add-on study, parallel group. Patients with flare of OA pain for 2-5 days were randomised to receive 1 or 2 Tram + apap tablets QID or placebo for 10 days in addition to ongoing NSAID or COX-2 SI.<br>Flare was defined as increased intensity of pain with need for additional analgesia or requiring increased NSAID dose.<br>QS=5	10 days	1 or 2 tablets of Tram 35 mg + para 325 mg QID (n=197)<br><br>Placebo (n=111)<br><br>Treatment with NSAID or Cox-2 SI continued	Whether or not related to treatment:<br>T + P 88/197 (45%)<br>Placebo 26/111 (23%)<br><br>Treatment related AEs:<br>T + P 48/197 (24%)<br>Placebo 9/111 (8%)	Dizziness<br>T + P 23/197 (12%)<br>Placebo 5/111 (5%)<br>Nausea<br>T + P 34/197 (17%)<br>Placebo 4111 (4%)<br>Vomiting<br>T + P 18/197 (9%)<br>Placebo 2/111 (2%)<br>Constipation<br>T + P 9/197 (5%)<br>Placebo 4111 (4%)<br>Darrhoea<br>T + P 6/197 (3%)<br>Placebo 5111 (5%)<br>Somnolence<br>T + P 14/197 (7%)<br>Placebo 2/111 (2%)<br>Pruritus<br>T + P 12/197 (6%)<br>Placebo 1/111 (1%)	T + P 25/197 (13%)<br>Placebo 6/111 (5%)<br><br>Most common with Tram/para: Nausea (8.6%), vomiting (5.6%), dizziness (4.6%), pruritis (2.5%)<br><br>Most common with placebo: headache (2.7%)<br><br>None were serious	T + P 1/197 (0.5%)<br>Placebo 0/111 (0%)
**Musculoskeletal pain**									
Arkinstall W et al. Efficacy of controlled-release codeine in chronic non-malignant pain: a randomized, placebo-controlled clinical trial. Pain 1995;62:169-178.	Codeine	Clinical trial	Random, double blind, placebo control, cross-over in 46 patients with chronic nonmalignant pain of at least moderate intensity (mainly rheumatic or back pain)<br>QS=4	7 days	Placebo (n=46)<br>Controlled release codeine200-400 mg daily (n=46)		Constipation<br>P 10% CR Cod 21%<br>Nausea<br>P 12% CR Cod 33%<br>Dizziness<br>P 14% CR Cod 21%<br>Somnolence<br>P 5% CR Cod 16%<br>Vomiting<br>P 5% CR Cod 14%<br>Pruritus<br>P 0% CR Cod 7%	Placebo 1/46 (2%)<br>CR Cod 7/46 (15%)	Placebo 0/46 (0%)<br>CR Cod 2/46 (4%)

Hale ME et al. Efficacy and safety of controlled-release versus immediate-release oxycodone: randomized, double-blind evaluation in patients with chronic back pain. Clin J Pain 1999; 15:179-83.	Oxycodone Controlled Release<br><br>Oxycodone Immediate Release	Clinical trial	Random, double blind (double dummy), active control, cross-over design, chronic low back pain (intevertebral disc disease & OA). Moderate or severe pain at study entry. Dose titration (open label): randomised to oxycodone CR 10 mg every 12 hrs or oxycodone IR 5 mgx4 daily. Titrated up from 20 mg daily until pain intensity <1.5 on a 4-point scale, total daily dose <80 mg. Then randomised to DB treatment for 4-7 days before cross-over to other treatment. No washout. Rescue medication wa oxycodone IR 5-10 mg. Nonopioid analgesics could be continued at stable doses. Adverse events were recorded. 50/57 previously on opioid / opioid combinations.<br>QS=4	4-7 days each cross-over	n=57 titration<br>n=47 DB phase<br><br>Period 1<br>Oxy CR 10 mg x 2 daily (n=25)<br><br>Oxy IR 5 mg x 4 daily (n=22)	Titration<br>O CR or IR 51/57 (89%)<br><br>DB phase<br>O CR or IR 36/47 (77%) in period 1<br>O CR or IR 29/57 (62%) in period 2	Period 1 only<br>Constipation<br>O CR 8/25 (32%)<br>O IR 10/22 (45%)<br>Dizziness<br>O CR 4/25 (16%)<br>O IR 2/22 (9%)<br>Nausea<br>O CR 4/25 (16%)<br>O IR 9/22 (41%)<br>Pruritus<br>O CR 7/25 (28%)<br>O IR 6/22 (27%)<br>Somnolence<br>O CR 3/25 (12%)<br>O IR 4/22 (18%)<br>Vomiting<br>none occurred	Titration phase:<br>6/57 (11%)<br>mainly nausea & vomiting<br><br>DB phase:<br>O CR 2/47 (4%)<br>O IR 1/47 (2%)	Titration phase:<br>2/57 (3.5%)
Moulin DE et al. Randomised trial of oral morphine for chronic non-cancer pain. Lancet 1996; 347:143-7.	1. SR Mo 60 mg x 2<br>2. Benztropine 1 mg x 2<br><br>(n=61)	Clinical trial	Random, double blind, placebo control, cross-over (2 wk washout). Musculoskeletal/ myofascial/ rheumatic regional pain, ≥ 6months duration; VAS ≥5/10, failure to respond to NSAIDs + one TCA. Excluded history of drug/alcohol abuse, previous strong opioids. Rescue paracetamol 500 mg (1 tablet given every 4 hrs). Previous opioids 60/61 were on codeine 120 mg/d prestudy. Daily diaries used to collect informaiton on adverse events.<br>QS=3	3 wk titration<br>6 wk evaluation<br>2 wk washout<br><br>Titration: 15, 30 and 60 mg tablets twice daily	(n=61)<br>Morphine SR 60 mg x 2<br>Benztropine 1 mg x 2<br><br>Mean daily dose:<br>M SR 83.5 mg<br>Benztropine 1.7 mg<br><br>120 mg, 20 pat:<br>60 mg, 22 pat:<br>130 mg, 4 pat	No data	Dose limiting<br>M SR 13/46 (28%)<br>Placebo 1/46 (2%)<br>Constipation<br>M SR 19/46 (41%)<br>Placebo 2/46 (4%)<br>Dizziness<br>M SR 17/46 (37%)<br>Placebo 1/46 (2%)<br>Poor appetite/nausea<br>M SR 18/46 (39%)<br>Placebo 3/46 (7%)<br>Vomiting<br>M SR 18/46 (39%)<br>Placebo 1/46 (2%)	Inadequate pain relief /AEs or both<br>Mo 11/61 (25%)<br>Placebo 4/61 (7%)	Inadequate pain relief /AEs or both<br>Mo 11/61 (25%)<br>Placebo 4/61 (7%)

Muller FO et al. Comparison of the efficacy and tolerability of a Paracetamol/Codeine fixed-dose combination with tramadol in patients with refractory chronic back pain. Arzneimittel-Forschung/Drug Research 1998; 48:675-9.	Codeine plus paracetamol<br><br>Tramadol	Clinical trial	Random, double blind, active control, cross-over design, fixed dose, refractory chronic back pain. Users of prolonged-life NSAIDs were given diclofenac 50 mg + misoprostol 200 ug 1-3 tablets daily as required at screening. All analgesics & anti-inflammatory drugs were discontinued before randomisation.Assessed at basline (day 1), global evaluation on day 8 & follow-up on day 15. Patients completed daily diaries. Adverse events were recorded - no further details.<br>QS=4	7 days each cross-over	Cod 30 mg + para 500 mg x 2 capsules (n=55)<br><br>Tramadol 50 mg x 2 capsules (n=55)<br><br>Drugs were given every 8 hr for 7 days	C + P 37/55 (69%)<br>T 37/55 (69%)	Constipation<br>C + P 15/55(27%)<br>T 0/55 (0%)<br>Dizziness<br>C + P 17/55 (31%)<br>T 18/55 (33%)<br>Dry mouth<br>C + P 9/55 (16%)<br>T 9/55 (16%)<br>Nausea<br>C + P 16/55 (29%)<br>T 16/55 (29%)<br>Pruritus<br>C + P 12/55 (22%)<br>T 15/55 (27%)<br>Somnolence<br>C + P 12/55 (22%)<br>T 15/55 (27%)<br>Vomiting<br>C + P 0/55 (0%)<br>T 11/55 (20%)	C + P 9/55 (16%)<br>T 10/55 (18%)	No data
Mullican WS, Lacy JR. Tramadol/acetaminophen combination tablets and codeine/acetaminophen combination capsules for the management of chronic pain: a comparative trial. Clin Ther 2001; 23(9):1429-45.	Tramadol + paracetamol<br><br>Codeine + paracetamol	Clinical trial	Random, double blind, active control, parallel groups, chronic lower back pain, OA or both. Mild (21%) to moderate pain. Excluded alcohol or drug abuse within 1 yr. Rescue medication - ibuprofen 400 mg every 4-6 hrs. Patient reports of adverse events whether or not related to study drug, spontaneously or in response to nodirected questioning. treatment-related = AE which emerged or worsened after initiation of drug therapy.<br>QS=5	4 wks	Tram + para (n=309)<br>Mean daily dose 3.5 tabs & caps 131 mg + 1133 mg (range 3-365 mg plus 28-3160 mg)<br><br>Cod + para (n=153)<br>Mean daily dose 3.5 tabs & caps 105 + 1054 mg, (range 9-253 mg plus 86-2534 mg)<br><br>Dose: 1 or 2 tablets & capsules every 4-6 hrs as needed. Max allowed 10 tabs / caps daily (8 if aged >75 yrs)<br><br>1 tab/cap:<br>Tram 37.5 mg + para 325 mg<br>Cod 30 mg + para 300 mg<br><br>80% completed the trial			T + P 27/309 (12%)<br>C + P 21/153 (14%)	No data
Orö L. A comparison between meptazinol and dextropopoxyphene plus paracetamol in elderly patients with musculoskeletal pains. Current Medical Researc and Opinion 1984;9:240-245.	Meptazinol<br>Dextropopoxyphene + paracetamol	Clinical trial	Random, double blind, active control, cross-over design, fixed dose, in patients with osteoporosis, osteoarthritis, lumbago or spondylosis in 31 patients.<br>QS=4	5 days	Meptazinol 800 mg daily (n=31)<br>DPP/paracetamol 260 + 2,600 mg (n=31)	M 3/31 (10%)<br>DPP/para 4/31 (13%)	Nausea<br>M 1/31 (3%)<br>DPP/para 2/31 (6%)<br>Dizziness<br>M 1/31 (3%)<br>DPP/para 2/31 (6%)	M 1/31 (3%)<br>DPP/para 1/31 (3%)	M 1/31 (3%)<br>DPP/para 2/31 (6%)

Ruoff GE. Slowing the initial titration rate of tramadol improves tolerability. Pharmacotherapy 1999;19:88-93.	Tramadol	Clinical trial	Randomised, double-blind, parallel group titration study. Tramadol dosing was additional to established NSAID therapy in patients with chronic joint pain<br>QS=3	14 days	Placebo (n=68)<br>1-day titation (n=130)<br>4-day titration (n=129)<br>10-day titration (n=132)<br>Titration to 200 mg tramadol daily		Dizziness<br>P 3/69 (4%)<br>1-day titation 31/132 (24%)<br>4-day titration 24/132 (19%)<br>10-day titration 11/132 (8%)<br>Constipation<br>P 2/69 (3%)<br>1-day titation 16/132 (12%)<br>4-day titration 14/132 (11%)<br>10-day titration 19/132 (14%)<br>Nausea<br>P 1/69 (2%)<br>1-day titation 37/132 (29%)<br>4-day titration 40/132 (31%)<br>10-day titration 27/132 (21%)<br>Vomiting<br>P 1/69 (2%)<br>1-day titation 13/132 (10%)<br>4-day titration 15/132 (12%)<br>10-day titration 11/132 (8%)<br>Somnolence<br>P 1/69 (2%)	P 3/69 (4%)<br>1-day titation 40/132 (31%)<br>4-day titration 31/132 (24%)<br>10-day titration 20/132 (15%)	P 0/69<br>1-day titation 1/132 (1%)<br>4-day titration 2/132 (2%)<br>10-day titration 2/132 (2%)
Schnitzer TJ et al. Efficacy of tramadol in treatment of chronic low back pain. J Rheumatol 2000; 27:772-8.	Tramadol<br><br>Placebo	Clinical trial	Enriched enrolment. Randomised, double blind, placebo controlled, parallel groups. Symptomatic, ambulatory patients with low back pain sufficient to require daily medication for ≥3 months. Maintained a constant level of activity throughout.<br>Screening/washout: 21 days<br>All pain medication, antidepressants, sedative hypnotics (except flurazepam or zolpidem tartrate), and anti-epileptic drugs given for pain control were discontinued before entry into the<br>Open-label run-in phase: 21 days<br>Patients with at least moderate pain were given tramadol, titrated up to 400 mg/day. Rescue medication (any short-acting analgesic) was allowed on Days 1-7 only. Assessments were made on Days 1, 7 and 21. On Day 21 patients were asked if the treatment	Titration 21 days<br>Double blind 4 wks	Tramadol (n=127)<br>Placebo (n=127)<br><br>Tramadol titrated up from 50 mg/day to 400 mg/day (maximum) over 21 days (open-label run-in). Double blind treatment for 28 days continuing the same dose as on Day 21 of the run-in.<br><br>Minimum daily dose of tramadol: 200 mg<br><br>Median days on treatment:<br>Tramadol: 29<br>Placebo: 18<br><br>Median dose/day (mg):<br>Tramadol: 250	Tram 43/127 (34%)<br>Placebo 26/127 (20%)	Nausea<br>Tram 11/127 (9%)<br>Placebo 3/127 (2%)<br><br>All particular adverse events occurred in fewer than 10% of patients in DB phase.<br><br>Open/DB phase:<br>nausea 17%, dizziness 14%, somnolence 14%, headache 12%	Titration phase:<br>Tram 78/380 (21%)<br><br>Double blind phase:<br>Tramadol: 5/127 (4%)<br>Placebo: 6/127 (5%)	Titration phase:<br>Tram 23/380 (6%)<br><br>Double blind phase:<br>Tramadol: 25/127 (20%)<br>Placebo: 61/127 (48%)

Sorge J, Stadler T. Comparison of the analgesic efficacy and tolerability of tramadol 100 mg sustained-release tablets and tramadol 50 mg capsules for the treatment of chronic low back pain. Clinical Drug Investiation 1997;14:157-164.	Tramadol	Clinical trial	Random, double blind, active control, parallel groups, chronic lower back pain.<br>QS=4	3 weeks	Tramadol 200 mg/day SR (2 x 100 mg) (n=103)<br>Tramadol 200 mg/day immediate release (4 x 50 mg)(n=102)	T SR 56/103 (54%)<br>T IR 54/102 (53%)	Particular adverse effects shown onl,y graphically, and were about the same in each group. Rates were approximately:<br>Nausea 16%<br>Dizziness 14%<br>Vomiting 10%<br>Fatigue 7%<br>Constipation 6%	T SR 15/103 (15%)<br>T IR 19/102 (19%)	
Thurel C, Bardin T et al. Analgesic efficacy of an association of 500 mg paracetamol plus 30 mg codeine versus 400 mg paracetamol plus 30 mg dextropropoxyphene in repeated doses for chronic lower back pain. Curr Ther Res 1991; 50: 463-73.j	Codeine plus paracetamol<br><br>Dextropropoxyphenepl us paracetamol	Clinical trial	Random, double blind, active control, parallel groups, chronic lower back pain.<br>QS=4	2 wks	Codeine 30 mg plus paracetamol 500 mg x 3 daily (n=25)<br><br>Dextropropoxyphene 30 mg plus paracetamol 400 mg x 4 daily (n=25)	C + P 2/25 (8%)<br>DP + P 4/25 (16%)	Upper gastrointestinal<br>C + P 7/25 (28%)<br>DP + P 5/25 (20%)<br>Constipation<br>"C + P 0/25 (0%)<br>DP + P 6/25 (25%)"	C + P 1/25 (4%)<br>DP + P 2/25 (8%)	
**Neuropathic pain**									
Harati Y, Gooch C, Swenson M et al. Double-blind randomized trial of tramadol for the treatment of the pain of diabetic neuropathy. Neurology 1998; 50:1842-6.	Tramadol<br><br>Placebo	Clinical trial	Random, double blind, placebo control, parallel groups, diabetic neuropathy. Washout (3 wks tricyclicss or anticonvusants, 1 wk analgesics).At least moderate pain. Excluded alcohol or narcotic abuse. Dose of tramadol titrated.<br>QS=4	42 days DB treatment	Tramadol 100-400 mg/day (n=65)<br><br>Placebo (n=66)<br><br>Titration:<br>Tramadol 50 mg/day on day 1, titrated up to 200 mg over 10 days. Max allowed days 14-28 was 400 mg/day.If inadequte relief, titration altered to max 400 mg/day by day 5. Min dose 100 mg/day. Patients remained on minimum effective dose for the rest of the study<br><br>Mean daily dose taken:<br>Tram 210 mg (SD 113)	No data	Constipation<br>Tram 14/65 (22%)<br>Placebo 2/66 (3%)<br>Dizziness<br>Tram 3/65 (5%)<br>Placebo 0/66 (0%)<br>Nausea<br>Tram 15/65 (23%)<br>Placebo 2/66 (3%)<br>Pruritus<br>Tram 4/65 (6%)<br>Placebo 0/66 (0%)<br>Somnolence<br>Tram 8/65 (12%)<br>Placebo 4/66 (6%)<br>Vomiting<br>Tram 3/65 (5%)<br>Placebo 0/66 (0%)	Tram 9/65 (14%)<br>Placebo 1/66 (2%)	Tram 9/65 (14%)<br>Placebo 22/66 (33%)
Harke H et al. The response of neuropathic pain and pain in complex regional syndrome I to carbamazepine and sustained-release morphine in patients pretreated with spinal cord stimulation: a double-blinded randomized study. Anesth Analg 2001;92:488-495.	**Phase 1:**<br>Carbamazepine<br>Placebo<br><br>**Phase 2:**<br>Morphine Sustained Release<br>Placebo	Clinical trial	Random, double blind, placebo control, parallel groups. Peripheral neuropathic pain reduced by SCS: post-discsurgeryradiculitis (17), PHN (6), phantom limb pain (3), diabetic neuropathy 83), periph nerve damage + CRPS (7); pain after inactivation of SCS (mean 7/10). No previous analgesics,or rescue analgesics allowed. 5 days dose adjustment, 7 days washout before phase 2. Previous opioids none immediately, before SCS 61% had been on weak opioid and 28% on strong opioids. Dropout rate in Phase 1 unclear.<br>QS=3	5-d dose adjustment, 8 days follow-up, 7 days washout before Phase 2	**Phase 1:**<br>CMZ 100 mg 2 x 3 (22)<br>Placebo 2 x 3 (n=21)<br><br>**Phase 2:**<br>Morphine SR 30 mg x 3 (n=21)<br>Placebo (n=17)<br><br>Final daily dose<br>Phase 1: CMZ 400-600 mg/d Phase 2:<br>M SR 60-90 mg/d (83 mg/d)	Data uninterpretable	**Phase 2:**<br>AE leading to dose reduction<br>M SR 8/21 (%)<br>Placebo 0/17 (0%)<br>Daily AEs<br>M SR 20/21 (%)<br>Placebo 2/17 (%)	No data	No data

Raja et al, 2002 (in press). Ask Eija where this will be published	Morphine (or methadone)<br>Nortriptyline (or desipramine)<br><br>If patients could not tolerate morphine or nortriptyline they were givent the alternative opioid or TCA (methadone, desipramine)	Clinical trial	Random, double blind, cross-over (1 wk washout). PHN for ≥ 3m, at least moderate of intensity (greater than 4 on 0-10 rating scale). Excluded history of substance abuse. All previous treatments for pain were stopped and no rescue medication allowed. previous opioids 23, TCA 22.<br>QS=5	2 week maintenance phase, 2-3 wks tapering, 1 week washout for all treatments<br><br>Flexible time period (typically 4 wks, range 1-9). Dose increased x 2 weekly until max PR achieved.<br><br>Max daily dose allowed 16 capsules: Morphine 240 mg, nortriptyline 160 mg	Morphine (or methadone)<br>Nortriptyline (or desipramine)<br><br>If patients could not tolerate morphine or nortriptyline they were givent the alternative opioid or TCA (methadone, desipramine)<br><br>Final daily dose:<br>Morphine 91 mg / day (range 15-225), n=38<br><br>Nortiptyline 89 mg/ day (40-140), n=46<br><br>Placebo 9 tablets / day<br><br>Methadone, n=26<br>Desipramine, n=13	Placebo / opioid / TCA<br>Constipation:<br>Placebo 2<br>Opioid 30<br>TCA 7<br>Nausea:<br>Placebo 4<br>Opioid 19<br>TCA 2<br>Dizziness:<br>Placebo 0<br>Opioid 18<br>TCA 17<br>Drowsiness:<br>Placebo 0<br>Opioid 18<br>TCA 7<br>Loss appetite:<br>Placebo 2<br>Opioid 5<br>TCA 2<br>Dry mouth:<br>Placebo 4<br>Opioid 7<br>TCA 6<br><br>Statistical significance:<br>Constipation & nausea opioids > placebo & TCA.<br>Dizziness TCA > placebo.<br>Drowsiness: opioid > placebo	Placebo / opioid / TCA<br>Constipation: 2/ 30/ 7<br>Nausea: 4 / 19/ 2<br>Dizziness: 0 / 18 / 17<br>Drowsiness: 0 / 18 / 7<br>Loss appetite: 2 / 5 / 2<br>Dry mouth: 4/ 7 / 6<br><br>Statistical significance:<br>Constipation & nausea opioids > placebo & TCA.<br>Dizziness TCA > placebo.<br>Drowsiness: opioid > placebo	No data	No data
Sindrup SH et al Tramadol relieves pain and allodynia in polyneuropathy: a randomised, double-blind, controlled trial. Pain 1999; 83:85-90.	Tramadol Slow Release<br><br>Placebo	Clinical trial	Random, double blind, placbo control, cross-over, allodynia in polyneuropathy. At least moderate pain (> 4/10 on VAS). One wk washout of pain medication. 1 wk for baseline observations before randomisation. Titrated dose of tramadol to maximum of 200 mg bid over a week. Dose reduced if adverse events unacceptable. Rescue analgesic: paracetamol 500 mg x6 daily. adverse events (dizziness, tiredness, dry mouth, sweating, constipation, micturition difficulty, nausea) were rated as mild or pronounced.<br>QS=4	4 wks each cross-over	n=45<br><br>Tramadol SR<br><br>Placebo<br><br>Max daily dose 400 mg/day	T SR 28/45 (62%)<br>Placbeo 12/45 (27%)<br><br>No relation between pain scores and adverse event scores	Constipation<br>T SR 10/45 (22%)<br>Placebo 2/45 (4%)<br>Dizziness<br>T SR 15/45 (33%)<br>Placebo 2/45 (4%)<br>Dry mouth<br>T SR 17/45 (38%)<br>Placebo 6/45 (13%)<br>Nausea<br>T SR 11/45 (24%)<br>Placebo 3/45 (7%)<br>Micturition difficulty<br>T SR 6/45 (13%)<br>Placebo 1/45 (2%)<br>Sweating<br>T SR 14/45 (31%)<br>Placebo 6/45 (13%)<br>Tiredness<br>T SR 19/45 (42%)<br>Placebo 4/45 (10%)	T SR 7/45 (16%)<br>Placebo 2/45 (4%)	None occurred

Watson CP et al. Efficacy of oxycodone in neuropathic pain: a randomized trial in postherpetic neuralgia. Neurology 1998; 50:1837-41.	Oxycodone Controlled Release<br><br>Placebo	Clinical trial	Random, double blind, placebo control, cross-over (no washout). PHN for ≥ 3m, at least moderate of intensity.Excluded history of drug/alcohol abuse. Previous antidepressants, NSAIDs and paracetamol were continued, no additional opioids. Titration at weekliy intervals to oxycodone 30 mg x 2 daily. Previous opioids45%. Results from last week of each treatment were compared, no washout. Adverse events collected using non-directed questionnaire.<br>QS=4	4wks x 2, no washout	(n=50)<br><br>Oxycodone Controlled Release 10-30 mg x 2<br><br>Placebo x 2<br><br><br>Final daily dose mean 45 ± 17 mg	No data	O CR:<br>Constipation 5/50 (10%)<br>Nausea 4/50 (8%)<br>Sedation 3/50 (6%)<br><br>No information for placebo	O CR 20-60 mg 5/50 (10%)<br>Placebo 3/50 (6%)	O CR 20-60 mg 0/50 (0%)<br>Placebo 1/50 (2%)
Mixed pain conditions									
Maier C & al. Morphine responsiveness, efficacy and tolerability in aptients with chronic non-tumour associateed pain - results of a double-blind placebo-controlled trial (MONTAS). Pain 2002; 97:223-233.	Morphine SR 10-90 mg x 2<br><br>Placebo	Clinical trial	Random, double blind, cross-over (no washout), palcebo control. Neuropathic/nociceptive pain >5/10 despite treatment. Previous non-opioids and co-analgesics were continued (step II opioids were stopped but could be used as rescue. 3-4 day titration, then 3 days evaluation.<br>Previous opioids Step 2 yes, Step 3 no.<br>Full responder: 50% pr/NRS <5/10, pain tolerability VRS <3,Aes tolerable.<br>Partial responder: PL response/pr on Mo insufficient/tolerable Aes, it was not possible to identify predictors of response; neuropathic>nociceptive, radiculopathy>low back, all 3 pancreatitis had full response; only 9% of the nearly 1000 screened patients met the inclusion criteria of severe pain after optimizing pretreatment<br>QS=4	3-4 days titration,<br>3 days evalution<br>no washout	(n=49)<br>M SR 10-90 mg x 2<br><br>Placebo<br><br>26 received morphine then placebo<br>23 received placebo then morphine<br><br>Median daily dose: 100 mg/d<br>38% of responders needed ≥ 120 mg/d<br><br>Permitted dose 60-180 mg/day	M SR 45/49 (92%)<br>Placebo 22/49 (45%)	Intolerable AEs<br>M SR 28/49 (58%)<br>Placebo 11/49 (22%)<br><br>Severe constipation<br>M SR 10/49 (20%)<br>Placebo 2/49 (4.5%)<br>Severe Nausea<br>M SR 16%<br>Placebo 2/49 (4.5%)<br>Severe Sedation<br>M SR 5/49 (10%)<br>Placebo 0/49 (0%)<br>Severe Dizziness<br>M SR 2/49 (4.5%)<br>Placebo 1/49 (2%)<br>Severe Pruritus<br>M SR 3/49 (6%)<br>Placebo 1/49 (2%)	3 patients	Placebo 2/49 (4.5%)<br>(placebo in phase 2)

Petrone D et al. Slowing the titration rate of tramadol HCl reduces the incidence of discontinuation due to nausea and/or vomiting: a double-blind randomized trial. Journal of Clinical Pharmacy & Therapeutics 1999; 24:115-23.	Tramadol	Clinical trial	Random, double blind, parallel groups, no control, chronic pain (musculoskeletal, neuropathic etc). NSAID continued throughout study. Open label dose titration, then randomsied to one of 3 groups Titrated up from 50 mg to 200 mg/day over 4 days. On 200 mg/day for 10 days then. Patients with nausea / vomiting could continue into DB phase after a 10 day washout. Randomised to 1 of 3 DB doseage groups (10, 13 or 16 day titration schedule). All adverse events were recorded & severity noted (mild, moderate, severe).<br>QS=4	4 weeks	Open label titration (n=931)<br><br>DB phase (n=167)<br>10-days to 200 mg tramadol (n=54)<br><br>16-days to 200 mg tramadol (n=59)<br><br>13-days to 150 mg tramadol (n=54)	Most adverse events were of mild-moderate intensity and resolved. They were less frequent in the 13 & 16 day titration groups<br><br>3 patients had a severe adverse event - none related to study treatment	Double blind phase<br>Constipation<br>10-days to 200 mg 4/54 (7%)<br>16-days to 200 mg 2/59 (3%)<br>13-days to 150 mg 6/54 (11%)<br>Dizziness<br>10-days to 200 mg 4/54 (7%)<br>16-days to 200 mg 4/59 (7%)<br>13-days to 150 mg 4/54 (7%)<br>Nausea<br>10-days to 200 mg 29/54 (54%)<br>16-days to 200 mg 25/59 (42%)<br>13-days to 150 mg 18/54 (33%)<br>Pruritus<br>10-days to 200 mg 2/54 (3%)<br>16-days to 200 mg 1/59 (2%)<br>13-days to 150 mg 4/54 (7%)<br>Somnolence<br>10-days to 200 mg 5/54 (9%)<br>16-days to 200 mg 4/59 (7%)	Open label titration 212/931 (%) due to nausea & / vomiting<br><br>DB phase<br>10-days to 200 mg 29/54 (54%)<br><br>16-days to 200 mg 20/59 (34%)<br><br>13-days to 150 mg 16/54 (30%)	DB phase<br>10-days to 200 mg 1/54 (2%)<br><br>16-days to 200 mg 2/59 (3%)<br><br>13-days to 150 mg 0/54 (0%)
Rauck RL et al. Comparison on tramadol and acetaminophen with codeine for long-term pain management in elderly patients. Current Therapeutic Research 1994;55:1417-1431.	Tramadol, Codeine + paracetamol	Clinical trial	Random, double blind, active control, with dose titration, and with restricted concomitant medication<br>QS=4	4 weeks	Tramadol (maximum 400 mg)(n=234)<br>Codeine + paracetamol (maximum 240/2,400 mg)(n=156)	Probably related to study drug<br>Patients<br>T 40/234 (17%)<br>CP 19/156 (12%)<br>Events<br>T 109<br>CP 50	Probably drug related<br>Nausea<br>T 24/234 (110%)<br>CP 7/156 (5%)<br>Vomiting<br>T 9/234 (4%)<br>CP 2/156 (1%)<br>Constipation<br>T 16/234 (7%)<br>CP 15/156 (10%)<br>Somnolence<br>T 14/234 (6%)<br>CP 5/156 (3%)<br>Dizziness<br>T 5/234 (2%)<br>CP 0/156 (0%)	T 44/234 (18%)<br>CP 15/156 (10%)	T 9/234 (4%)<br>CP 10/156 (6%)
