# Supplementary material for: Prevalence of opioid adverse events in chronic non-malignant pain: systematic review of randomised trials of oral opioids
Source: Arthritis Res Ther. 2005 Jun 28;7(5):R1046–51. doi: 10.1186/ar1782 (PMC1257433; doi:10.1186/ar1782)
Supplement: Additional File 2 — A pdf file which lists the adverse events with opioids in different pain conditions. [file ar1782-S2.pdf]

Appendix 2: Adverse events with opioids in different pain conditions

Adverse events with opioids in arthritis

	Number of		Event rate (%)		
**Adverse event**	**Trial arms**	**Patients**	**Average**	**95% CI**	**Range**
Patients experiencing any AE	15	1187	54	51-57	35-87
**Withdrawals**					
Adverse event withdrawal	24	2173	26	25-28	2-71
Lack of efficacy withdrawal	16	1416	7.8	6.4-9.2	0-40
**Specific adverse events**					
Dry mouth	8	399	25	21-29	3-70
Nausea	20	1864	24	22-26	9-98
Consitipation	21	1915	18	16-20	2-71
Dizziness	18	1809	14	13-16	5-33
Drowsiness or somnolence	13	1092	13	11-15	2-39
Pruritus	5	356	15	11-18	6-38
Vomiting	17	1396	13	11-15	2-23

Adverse events with opioids in musculoskeletal pain

	Number of		Event rate (%)		
**Adverse event**	**Trial arms**	**Patients**	**Average**	**95% CI**	**Range**
Patients experiencing any AE	12	1063	57	55-61	8-77
**Withdrawals**					
Adverse event withdrawal	14	1331	16	14-18	4-30
Lack of efficacy withdrawal	7	631	5.7	3.9-7.5	0.8-20
**Specific adverse events**					
Dry mouth					
Nausea	16	1504	21	19-23	9-39
Consitipation	15	1362	13	11-15	0-46
Dizziness	15	1374	17	15-19	8-37
Drowsiness or somnolence	11	1220	18	16-20	7-30
Pruritus	4	157	26	19-32	22-28
Vomiting	13	1312	9.7	8-11	0-39

Adverse events with opioids in neuropathic pain

Adverse event	Number of		Event rate (%)		
	Trial arms	Patients	Average	95% CI	Range
Patients experiencing any AE	1	45	62	48-76	
**Withdrawals**					
Adverse event withdrawal	3	160	13	7.9-18	10-16
Lack of efficacy withdrawal	2	115	7.8	2.9-13	0-14
**Specific adverse events**					
Dry mouth					
Nausea	3	160	19	13-25	8-24
Consitipation	3	160	18	12-24	10-22
Dizziness	2	110	16	9.5-23	5-33
Drowsiness or somnolence	3	160	19	13-25	6-42
Pruritus	1	65	6	0.3-12	
Vomiting	0	65	0		

Adverse events with opioids in pain of mixed origin

Adverse event	Number of		Event rate (%)		
	Trial arms	Patients	Average	95% CI	Range
Patients experiencing any AE	3	439	24	20-28	17-92
**Withdrawals**					
Adverse event withdrawal	5	557	22	19-26	10-54
Lack of efficacy withdrawal	5	557	3.9	2.3-5.6	0-6.4
**Specific adverse events**					
Dry mouth					
Nausea	6	606	18	15-24	4.5-54
Consitipation	6	606	8.7	6.5-11	3.4-20
Dizziness	6	606	3.1	1.7-4.5	0-7.4
Drowsiness or somnolence	6	606	5.4	3.6-7.3	0-10
Pruritus	4	216	4.6	1.8-7.4	1.7-7.4
Vomiting	5	557	5.7	3.8-7.7	1.3-19

Note: RCTs only, any comparator, any dose of opioid, any opioid, any combination, trials to 8 weeks.
